# Limbal stem cell deficiency secondary to systemic paclitaxel (Taxol) for breast cancer: a case report

**DOI:** 10.1186/s12886-020-01672-x

**Published:** 2020-10-07

**Authors:** Amardeep Sekhon, Jeff Y. F. Wang, Johnson C. H. Tan, Simon P. Holland, Sonia N. Yeung

**Affiliations:** 1 Department of Anesthesiology, Pharmacology and Therapeutics, Faculty of Medicine, University of British Columbia, Vancouver, Canada; 2 Faculty of Medicine, University of British Columbia, Vancouver, Canada; 3 Division of Cornea and External disease, Department of Ophthalmology and Visual Sciences, University of British Columbia, Vancouver, Canada; 4 National Healthcare Group Eye Institute, Tan Tock Seng Hospital, Singapore, Singapore; 5 Pacific Laser Eye Centre, Vancouver, Canada; 6 Eye Care Centre, Vancouver Hospital, 2550 Willow Street, Vancouver, BC V5Z 3N9 Canada

**Keywords:** Limbal stem cell deficiency, Paclitaxel, Chemotherapy, Breast cancer, Keratitis

## Abstract

**Background:**

Paclitaxel (PTX) is an antineoplastic drug widely used in treatments for ovarian, breast, and small-cell lung cancer. Although ocular effects associated with PTX have been previously described, very few studies have specifically reported systemic PTX as a contributing factor for limbal stem cell deficiency (LSCD), which is characterized by the loss of stem cell and barrier function of the limbus leading to progressive pain and reduction in visual acuity. Described here is a unique case where a patient was diagnosed with LSCD secondary to PTX use for the treatment of breast cancer, at doses of PTX far lower than what is reported in current literature.

**Case presentation:**

A 73-year-old woman with a previous diagnosis of breast cancer with liver metastasis presented with a complaint of increasing pain in the left eye more than the right, along with decreasing visual acuity in both eyes following 3 months of PTX therapy for recurrent liver metastases. Upon examination, best-corrected visual acuity was 20/100 in the right eye and counting fingers on the left. Peripheral neovascularization, stromal scarring, and features of limbal stem cell deficiency (LSCD) were noted on the right cornea. A central neurotrophic ulcer with thinning to 50% and 360 degrees of conjunctivalization were noted on the left. After the discontinuation PTX with doxorubicin as the substitute, there was no further progression of her LSCD, and stabilization of her ocular surface was achieved.

**Conclusion:**

Although chemotherapy induced LSCD is a relatively rare adverse event, it is essential for clinicians starting new chemotherapy agents to consider the potential ocular toxicities that may result in their use. Ophthalmology review is recommended for patients after starting PTX therapy to assess for signs of LSCD, particularly in patients where drug toxicity can be aggravated due to impaired hepatic function.

## Background

Limbal stem cell deficiency (LSCD) is an uncommon but serious condition where the stem cell and barrier function of the limbus is lost leading to progressive pain and reduction in vision [1]. Loss of limbal stem cells results in an epitheliopathy with an unstable ocular surface, chronic keratitis and neovascularization inevitably leads to conjunctivalization and scarring of the cornea. The gold standard of diagnosis is impression cytology [1], although most cases are diagnosed clinically. Although ocular toxicity associated with Paclitaxel (PTX)(Taxol) has been previously described, few studies have specifically reported systemic PTX as a contributing factor for limbal stem cell deficiency (LSCD). However, various studies have reported various ocular toxicities such as cystoid macular edema occurring from PTX therapy for breast and ovarian cancer treatment [2, 3]. A case of LSCD in a patient with no previous ocular history treated with high-dose PTX monotherapy for breast cancer has previously been reported [4]. We herein report a patient with LSCD secondary to PTX use for the treatment of recurrent liver metastases at doses far lower than used in similar cases in the current literature, where the effect of PTX at lower doses may have been compounded by her past medical history.

## Case presentation

**Fig. 1:**
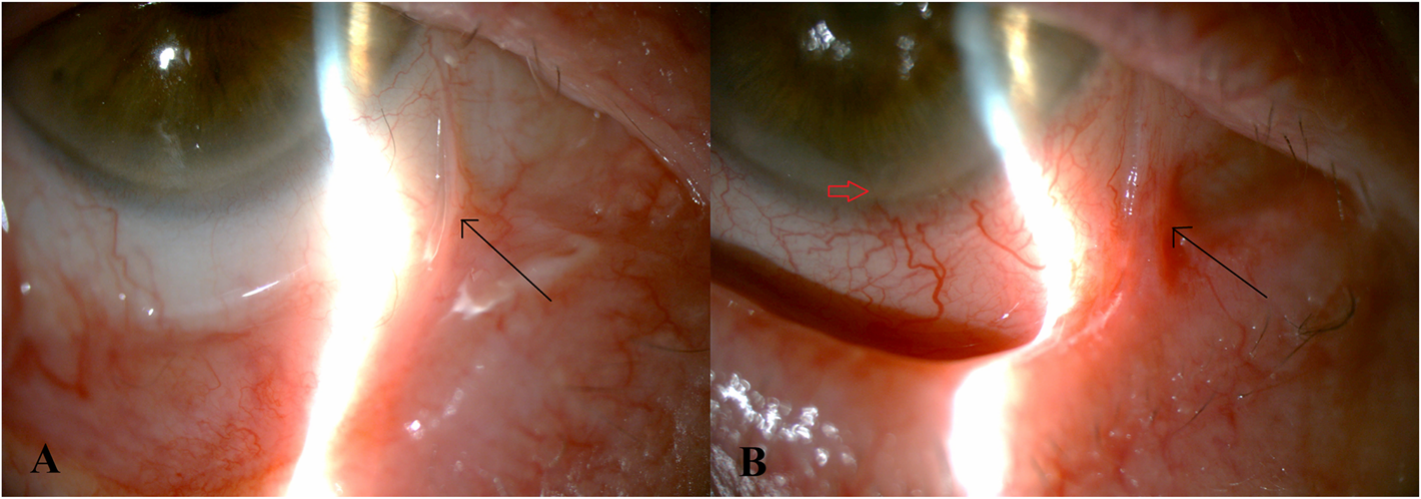
**a**, Infero-nasal symblepharon in the right eye (black arrow). **b**, Temporal symblepharon in the left eye (black arrow), with peripheral corneal vascularization (red arrow)

**Fig. 2:**
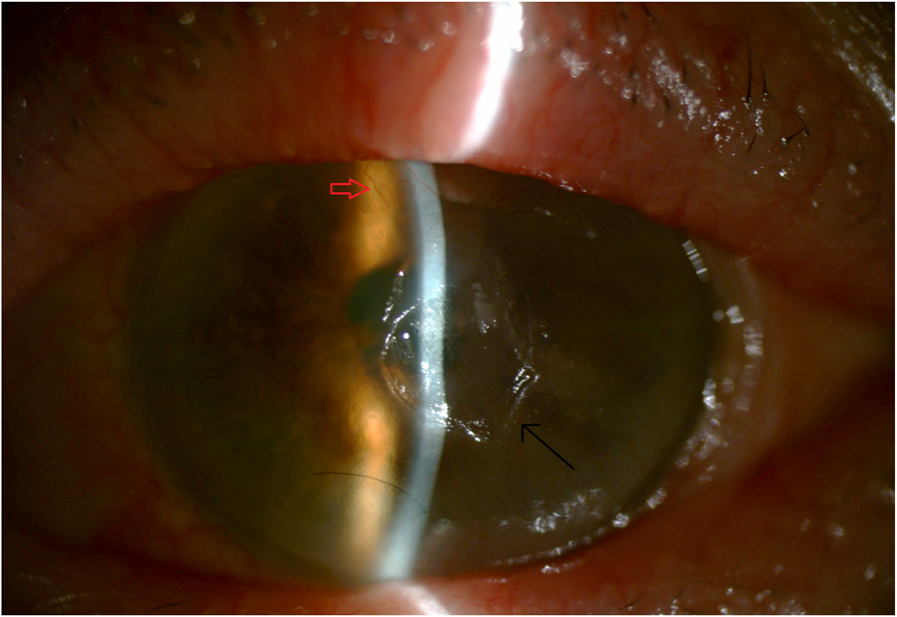
Central neurotrophic ulcer (1.4mmx2.8 mm) with corneal thinning (black arrow) in the left eye. Superior corneal vascularization is evident (red arrow)

A 73-year-old woman with a history of breast cancer with liver metastasis presented with increasing pain in the left eye more than the right, associated with increasing blurring of vision in both eyes 3 months after beginning PTX. She was diagnosed with chronic posterior blepharitis associated with mild symblepharon formation 7 years ago. Her ocular history was otherwise unremarkable. Mild inferior pannus was noted and there was no evidence of LSCD. No lagophthalmos or lid abnormalities was noted in either eye. Breast cancer was diagnosed 3 years ago and liver metastases less than 1 year ago. She was initially started on doxorubicin and cyclophosphamide for 12 weeks, then docetaxel and trastuzumab for the next 12 weeks with dosing every 3 weeks. She responded well to treatment and was carried on with trastuzumab for another 52 weeks. For the past year she has been taking exemestane 25 mg daily, however given a recurrence of her breast cancer as liver metastases, she was started on weekly PTX 80 mg/m^2^ and trastuzumab 8 mg/kg. While on her third month of PTX with good response, she presented to the Cornea Service with decreasing vision associated with redness and pain. Her past medical history included non-insulin dependent diabetes mellitus well-controlled on metformin. She had never been at soft contact lens wearer. Upon examination, best-corrected distance visual acuity was 20/100 in the right and counting fingers in the left eye. Bilateral stable temporal and nasal small symblephara were noted since her initial visit in 2006 (Fig. 1a, b). Blunting of the corneal sensation was noted on the left. Peripheral neovascularization, peripheral stromal scarring, and a whorled epitheliopathy staining late with fluorescein consistent with LSCD from 10 to 5 o’clock were present on the right cornea. A central neurotrophic ulcer measuring 1.4mmx2.8 mm, with central stromal haze and thinning to 50%, and complete LSCD with 360 degrees of conjunctivalization staining late with fluorescein were noted on the left cornea (Fig. 2). Intraocular pressures were 14 in both eyes. There were mild nuclear sclerotic lens changes noted bilaterally.

Differential diagnoses for LSCD in this patient included chronic posterior blepharitis, paraneoplastic ocular cicatricial pemphigoid (OCP) and LSCD secondary to PTX toxicity. LSCD secondary to other ocular surface disorders such as neurotrophic keratopathy has been reported. Given that there was blunting of the corneal sensation in the left eye, this factor must also be considered [5]. A large conjunctival biopsy showed chronic inflammation and fibrosis, with no pathological or immunohistochemical features of OCP. Her chronic blepharitis had been documented to be under control and stable without significant flares over the years.

A provisional diagnosis of LSCD secondary to PTX was made. She was started on a regimen of preservative-free artificial tears, preservative-free prednisolone (0.5%) eyedrops 4 times daily, and autologous serum tears 4 times daily. A temporary lateral tarsorrhaphy performed 2 weeks later did not reduce the non-healing epithelial defect over another 2 weeks. Thus, PTX was discontinued by her oncologist and she was switched to doxorubicin. A month later, the epithelial defect was completely healed although her vision remained at count fingers on the left due to central corneal scarring. The tarsorrhaphy was subsequently released, and her ocular surface remained stable over 19 months. The vision and extent of LSCD remained unchanged on the left. The right eye showed recovery of best corrected vision to 20/30 with only mild peripheral pannus noted.

## Discussion and conclusions

The use of both topical ocular and systemic chemotherapy drugs has been linked to LSCD [6–8], but few studies [4] have specifically reported systemic PTX as a contributing factor for LSCD. LSCD resulting from subconjunctival injection of 5-fluorouracil (5-FU) have also been reported [9]. Since limbal stem cells reside within the palisades of Vogt, which are crypts in the basal layer of the limbal epithelium [10], topical chemotherapy agents like mitomycin C and 5-FU may penetrate through the corneal epithelial layers in order to affect these cells by alkylating DNA, and inhibiting DNA and RNA synthesis [11]. The limbus is also well vascularized, allowing systemic chemotherapy to produce its toxic effects on limbal stem cells [1]. Hydroxycarbamide-induced LSCD was reported at 2 years after initiation for chronic myelocytic leukemia at 1500 mg daily [6] and noted in another case at 4 years after initiation for pulmonary hypertension at 500/1000 mg alternating daily [8]. These cases were both treated significantly longer than our patient who had only been using PTX for 3 months.

Despite having been on other chemotherapeutic agents, there are several factors pointing towards PTX as the etiology in our patient. The weekly dosing of PTX was more aggressive compared to her initial chemotherapy treatment regimen years ago in light of liver metastasis. Our patient also had liver failure secondary to liver metastases, which can lead to PTX toxicity and worsening of its associated side effects [12]. Although we cannot be certain that her previous chemotherapy regimen did not have any role in her disease, she had been followed regularly for chronic blepharitis, and her ocular symptoms and clinical appearance of LSCD did not appear until after starting the current PTX regimen. Furthermore, for the past year, she was only on exemestane, an aromatase inhibitor that blocks the synthesis of estrogen. There have been no reported cases of LSCD after using doxorubicin, cyclophosphamide, docetaxel or trastuzumab. On follow up examination after discontinuing PTX and restarting her on doxorubicin, there was no further progression of her LSCD, and stabilization of her ocular surface was achieved.

PTX is an anti-microtubule agent that promotes microtubule assembly and stabilizes it from disassembly thus producing its cytotoxic effects on cells [13]. Pharmacokinetically, PTX is a hydrophobic drug and can distribute to the peripheral compartments [14] such as the limbal basal layer. In rabbit studies where limbal based conjunctival flaps were created, topical mitomycin C and topical PTX both reduced fibrosis and cell counts equally, thus the toxic effects of PTX can be just as potent as mitomycin C [15].

Like other chemotherapeutic drugs, PTX affects rapidly growing cells more than quiescent cells. Limbal stem cells are generally quiescent; however, rapid growth of limbal stem cells can be induced by corneal injuries in order to re-epithelialize the damaged cornea [1]. Our patient’s chronic blepharitis was likely a chronic inciting factor, causing persistent inflammatory damage to her cornea and lowering the threshold for LSCD. With an increased basal rate of division in limbal stem cells, it is conceivable that PTX can potentiate significant LSCD in a short period of 3 months. A study done by Lee [4] reported a breast-cancer patient with bilateral corneal epithelial lesions resulting from paclitaxel monotherapy, where a diagnosis of LSCD secondary to paclitaxel use was made. However, this patient had no significant ocular history that could have been an aggravating factor for developing LSCD compared to our patient. Furthermore, dosing in Lee’s study, which was 254.54 mg/m^2^, was much higher than that of our patient’s, which was 80 mg/m^2^. This shows that although our patient had received a much lower dose of paclitaxel to treat the recurrence of liver metastases, the ocular toxicities of paclitaxel may have been compounded with our patient’s history of chronic blepharitis and impaired liver function, lowering the threshold for LSCD.

Although chemotherapy induced LSCD is relatively rare, prompt action should be taken to avoid long term ocular and visual morbidity. Ellies [6] reported that stopping hydroxycarbamide led to reversal of LSCD and other epithelial defects in one eye while the other did not fully improve. LSCD should not be the only consideration of the clinician; it is worth noting that in some cases [16] where PTX was considered the etiology in a non-LSCD ocular disorder, the effects of PTX can be irreversible leading to permanent loss of vision. Several studies [2, 3, 16, 17] have reported other ocular toxicities resulting from PTX use, with cystoid macular edema being the most commonly reported side-effect. At our patient’s last follow-up, 19 months following cessation of PTX, her ocular surface remained stable.

It is recommended that patients with ocular surface inflammatory changes compounded by factors that prevent drug clearance, such as hepatic failure, to have ophthalmological follow up after starting new chemotherapy agents to assess for signs of LSCD particularly in the setting of decreasing vision. Although ocular disorders caused by PTX are relatively rare adverse events, it is essential for clinicians starting new chemotherapy agents to consider the potential ocular toxicities that may result in their use and arrange for appropriate ophthalmological review.

## Data Availability

Not Applicable.

## Abbreviations

LSCD: Limbal stem cell deficiency
PTX: Paclitaxel
OCP: Paraneoplastic ocular cicatricial pemphigoid
5-FU: 5-fluorouracil
